# Cutaneous microbial biofilm formation as an underlying cause of red scrotum syndrome

**DOI:** 10.1186/s40001-021-00569-9

**Published:** 2021-08-19

**Authors:** Theodore W. Perry

**Affiliations:** Fee-Basis Hospitalist, North Texas VA Medical Center, 4500 South Lancaster Road, TX 75216 Dallas, USA

**Keywords:** Red scrotum syndrome, Cutaneous microbial biofilm, Tinea versicolor, Vulvodynia

## Abstract

**Background:**

Red scrotum syndrome is typically described as well-demarcated erythema of the anterior scrotum accompanied by persistent itching and burning. It is chronic and difficult to treat and contributes to significant psychological distress and reduction in quality of life. The medical literature surrounding the condition is sparse, with the prevalence likely under-recognized and the pathophysiology remaining poorly understood. Formation of a cutaneous microbial biofilm has not been proposed as an underlying etiology. Microbial biofilms can form whenever microorganisms are suspended in fluid on a surface for a prolonged time and are becoming increasingly recognized as important contributors to medical disease (e.g., chronic wounds).

**Case presentation:**

A 26-year-old man abruptly developed well-demarcated erythema of the bilateral scrotum after vaginal secretions were left covering the scrotum overnight. For 14 months, the patient experienced daily scrotal itching and burning while seeking care from multiple physicians and attempting numerous failed therapies. He eventually obtained complete symptomatic relief with the twice daily application of 0.8% menthol powder. Findings in support of a cutaneous microbial biofilm as the underlying etiology include: (1) the condition began following a typical scenario that would facilitate biofilm formation; (2) the demarcation of erythema precisely follows the scrotal hairline, suggesting that hair follicles acted as scaffolding during biofilm formation; (3) despite resolution of symptoms, the scrotal erythema has persisted, unchanged in boundary 15 years after the condition began; and (4) the erythematous skin demonstrates prolonged retention of gentian violet dye in comparison with adjacent unaffected skin, suggesting the presence of dye-avid material on the skin surface.

**Conclusion:**

The probability that microorganisms, under proper conditions, can form biofilm on intact skin is poorly recognized. This case presents a compelling argument for a cutaneous microbial biofilm as the underlying cause of red scrotum syndrome in one patient, and a review of similarities with other reported cases suggests the same etiology is likely responsible for a significant portion of the total disease burden. This etiology may also be a significant contributor to the disease burden of vulvodynia, a condition with many similarities to red scrotum syndrome.

## Introduction/background

Red scrotum syndrome (RSS) is typically described as well-demarcated scrotal erythema accompanied by symptoms of persistent itching and burning. It affects the anterior portion of the scrotum, sometimes with extension to the inferior and posterior portions and to the ventral shaft of the penis [1–3]. Affected patients are commonly age > 50, though young patients are also affected. The condition is typically chronic and difficult to treat, with symptoms often lasting for years and contributing to significant psychological distress and reduction in quality of life [4]. The medical literature surrounding RSS is sparse, and the prevalence of the condition is likely under-recognized. Primary neuropathy, microvascular dysregulation, and overuse of topical corticosteroids have been suggested as potential causes, but the underlying pathophysiology remains poorly understood [5, 6]. It is possible the condition represents a constellation of similar symptoms and physical findings that result from a number of different etiologies. This report describes one patient’s 15-year experience with the condition, and provides evidence implicating a cutaneous microbial biofilm as the likely etiology. In addition, a review of the current literature will implicate the same etiology as likely responsible for a significant portion of the total RSS disease burden.

In nature, microorganisms (e.g., bacteria, fungi) exist in 2 states: planktonic (free in the environment) and within biofilm (surface-attached). The biofilm state can occur whenever microorganisms are suspended in fluid on a surface. Biofilm is created when microbes attach to the surface and subsequently secrete extracellular polymers that form a protective matrix, allowing the microorganisms to live and grow in protection from environmental threats [7–10]. Evidence shows that under proper conditions biofilm formation can take place within several hours, with continued maturation occurring over days, months, and years [11]. Once created, biofilms are notoriously difficult to eradicate, as the microorganisms are protected from antibiotics and other chemical antimicrobials by the thick extracellular matrix. In addition, microbes within biofilm often assume a relatively slow growth and metabolic rate, creating less susceptibility to toxic inhibitors. Microorganisms within biofilm also have the ability to efficiently communicate with one another (i.e., quorum sensing) and rapidly share extrachromosomal genetic material, further amplifying their capacity for antimicrobial resistance [12–14].

Over the past 20 years, microbial biofilms have been increasingly recognized for playing a prominent role in human infection. Early on, evidence primarily implicated biofilms in infections that involve nonliving surfaces, including urinary or intravenous catheters, prosthetic joints, and prosthetic heart valves [15]. However, microbial biofilms have more recently been appreciated as either significant contributors or the underlying cause of many difficult-to-treat and/or chronic diseases that involve biofilm attachment to living surfaces. Among these conditions are native valve infective endocarditis, chronic prostatitis, chronic wound infections, and chronic sinusitis [16–19]. Undoubtedly, the role of microbial biofilms in other chronic conditions is yet to be recognized.

## Case presentation

At condition onset, the patient was a 26-year-old man with a history of recurrent tinea versicolor affecting the trunk and neck and no other significant medical history, including no history of topical corticosteroid use. He experienced the abrupt onset of erythema of the bilateral scrotum accompanied by intense itching and burning. The signs and symptoms began one morning following sexual intercourse with a female partner the previous night. The patient did not clean up after intercourse and fell asleep lying on his back with vaginal secretions covering his scrotum.

**Fig. 1:**
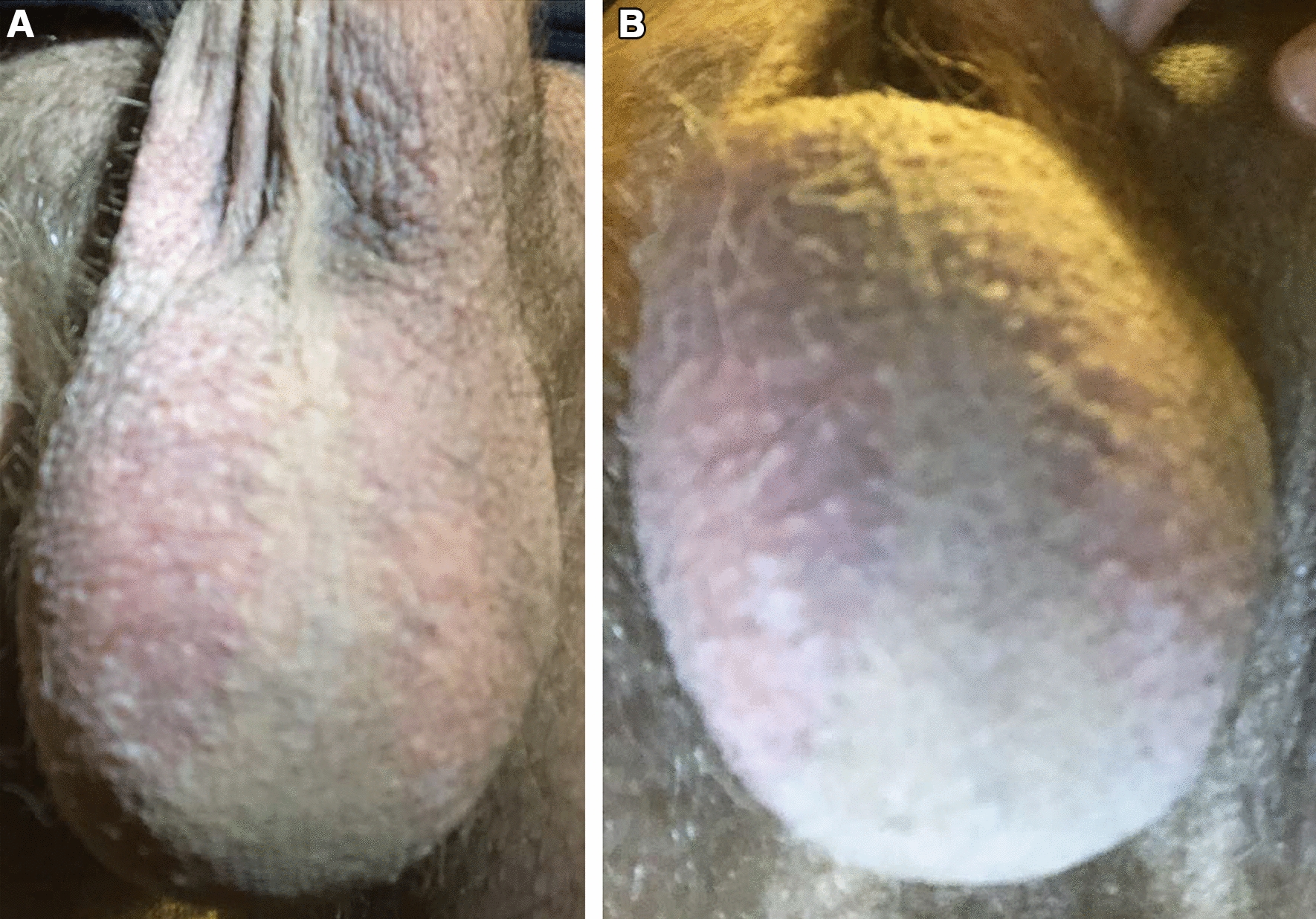
**A** Photograph of the anterior scrotum demonstrating well-demarcated bilateral erythema with sparing of the midline. The erythema is seen to closely follow the boundary of hair follicles (scrotal hair has been trimmed). **B** Photograph taken from a slightly different angle demonstrating the unaffected underside of the scrotum.

On examination, the erythema was well-demarcated, closely following the distribution of hair on the bilateral scrotum and also involving a small portion of the hair-covered ventral shaft of the penis. There was sparing of erythema along the hairless portion of the scrotal midline and the hairless underside of the scrotum (Fig. 1). There was no evidence of ulceration or swelling, and there was no tenderness on palpation of the affected area. The patient first sought care with his primary care physician, where HIV testing and HSV 1 and 2 serum antibody testing were performed and were all negative. The patient then completed a 4-week course of topical clotrimazole cream with no improvement in symptoms. A 10-day course of erythromycin was also tried under the assumption that the condition represented an atypical presentation of erythrasma, but also with no improvement.

Symptomatically, the patient continued to experience nearly constant symptoms of scrotal itching and burning that were made worse by long periods of sitting. He was subsequently referred to a dermatologist at a large academic center, where he was told the condition represented some type of mast cell overactivity and there was no specific treatment for it. Unsatisfied with that conclusion, the patient returned to see another dermatologist at the same institution. After a brief examination, the second dermatologist told the patient his scrotal skin was “normal,” and he should try to “forget about” his symptoms. At the insistence of the patient, a punch biopsy of the erythematous scrotal skin was performed, with the dermatopathologic results reported as normal other than hypervascularity.

During this time the patient experienced significant psychological distress, including decreased concentration, insomnia, anhedonia, decreased libido, and decreased appetite with weight loss. Seeking further assistance, the patient sought the opinion of a private practice dermatologist, who had previous experience as a military physician. That dermatologist acknowledged that he had seen about a dozen cases of RSS previously and advised that from his experience only about half of patients experience improvement. Additional treatments were attempted including pulsed dye laser therapy (targeted at hypervascularity) and liquid nitrogen cryotherapy, neither of which provided improvement. An extended course of minocycline was also attempted, but the patient had to discontinue the drug after several days due to intolerance (metallic taste).

After 14 months with no improvement in symptoms and only slight fading of the erythema, the patient began applying 0.8% menthol powder (e.g., extra strength Gold Bond) to the scrotum twice per day after cleaning the skin with water and thorough drying with a towel. The itching and burning symptoms gradually improved and eventually resolved after 1–2 months of treatment. The erythema persisted, however, slightly faded but without change in affected area or demarcation. Over time, the patient was able to wean down to once daily application of 0.8% menthol powder, and he has continued this regimen for over 14 years, remaining mostly symptom free. The intense itching and burning has briefly returned on a few occasions, most notably during a 2-week-long period (12 years after the condition first began) when the patient ingested 8 oz of kefir daily in an effort to improve gastrointestinal upset. The patient eventually suspected the kefir as an exacerbating factor and discontinued use, with the itching and burning again resolving within a couple weeks.

**Fig. 2:**
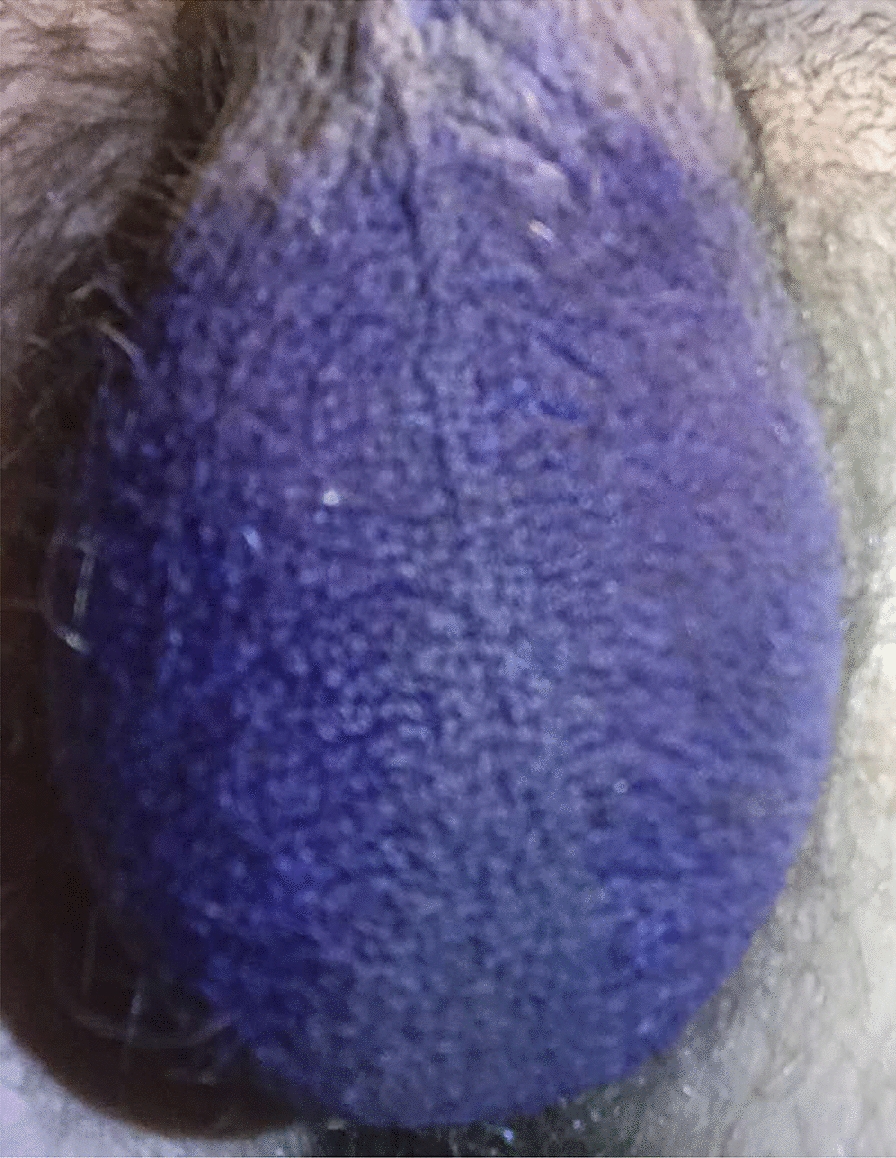
Photograph demonstrating scrotal appearance at the time of gentian violet application.

**Fig. 3:**
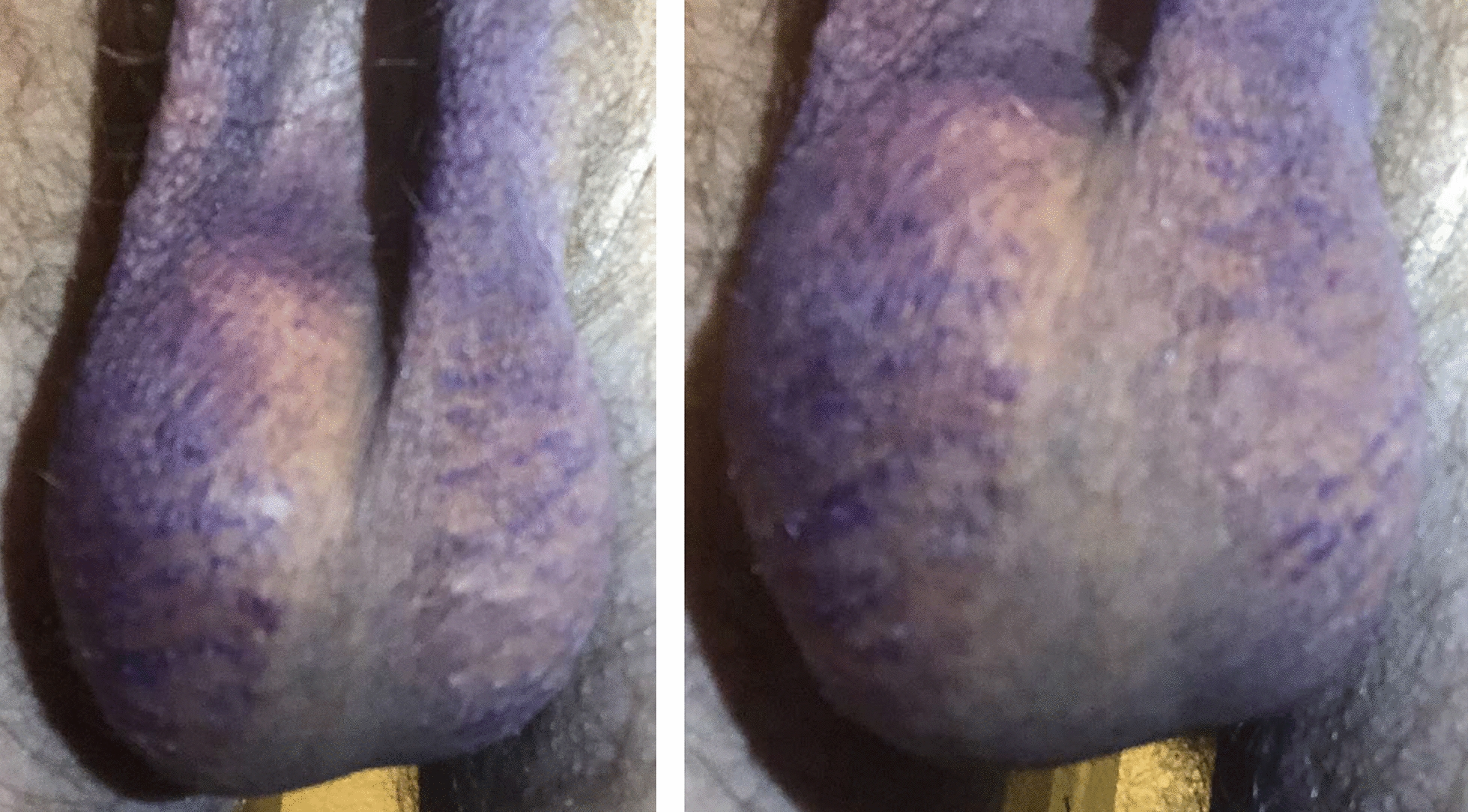
Two photographs taken from slightly different angles 1 day after gentian violet application (after the patient has showered once). Dye remains on only the affected skin, closely following the borders of erythema.

**Fig. 4:**
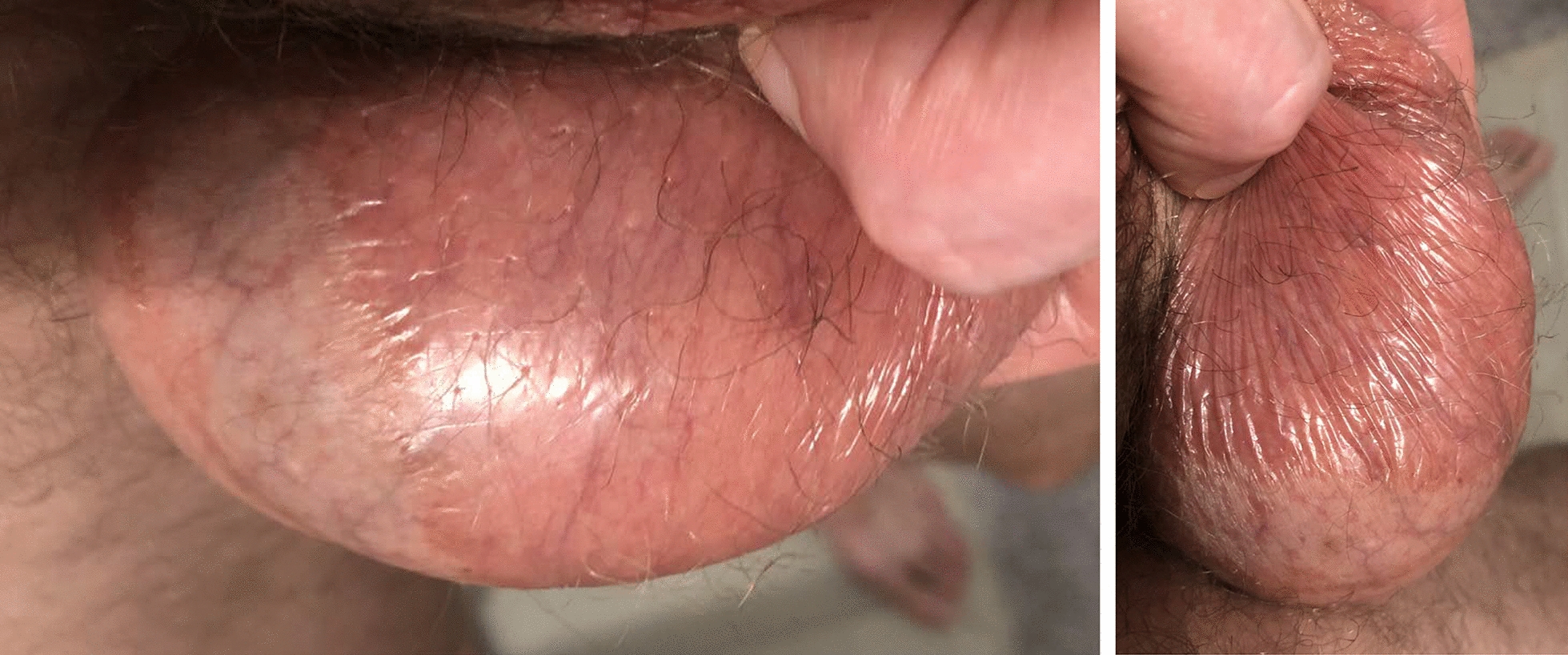
Photographs of the right side of the scrotum after 2–3 days of twice daily application of 2.5% selenium sulfide solution. The affected skin has become slightly raised and glassy-appearing, in sharp contrast with the unaffected scrotal midline.

**Fig. 5:**
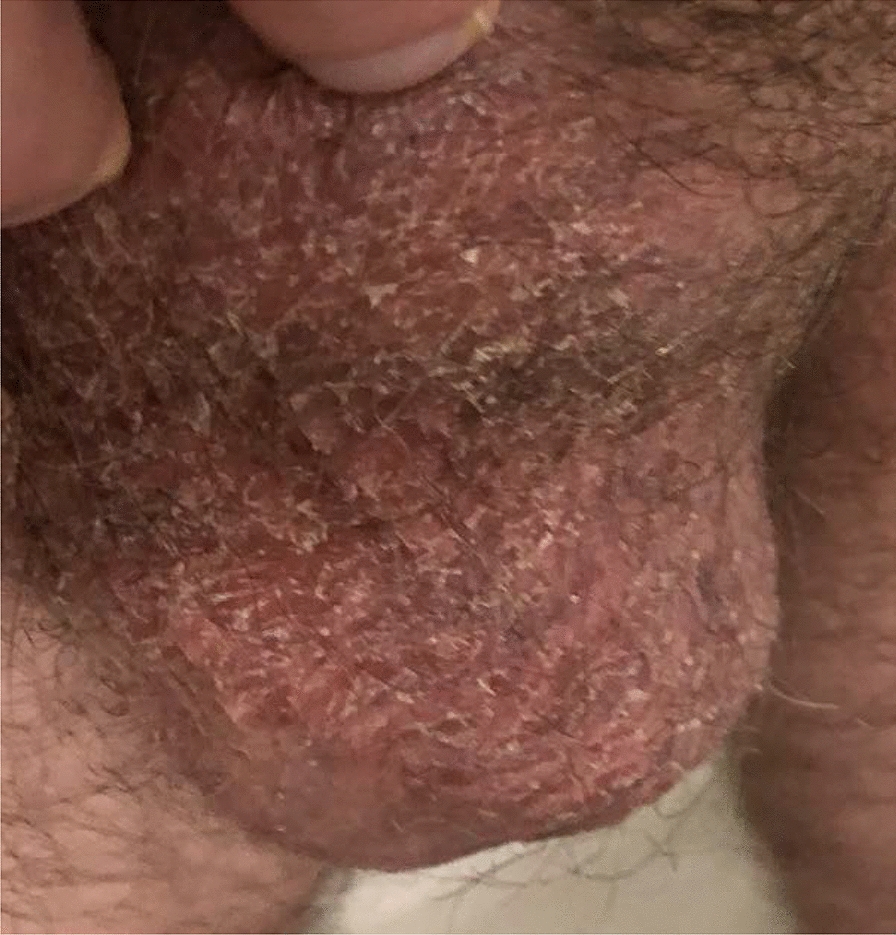
Photograph of the left side of scrotum after approximately 1 week of twice daily application of 2.5% selenium sulfide solution, showing that the affected skin has become desiccated and flaking. A small area of the unaffected scrotal midline is seen just below and to the patient’s right of the affected skin.

Over the years, the patient has attempted other treatments in an effort to completely eradicate the condition. At one point, he attempted daily application of gentian violet to the affected area given evidence of antistaphylococcal and antifungal properties of the agent. The therapy was discontinued after several days due to worsening scrotal discomfort. Remarkably, however, the gentian violet quickly washed off of the unaffected scrotal skin but for several days remained in place on the affected skin, exactly matching the erythematous borders to suggest the presence of something holding it there (Figs. 2 and 3). The twice daily application of 2.5% selenium sulfide solution was also attempted. After 2–3 days of therapy, the affected scrotal skin became bright red and slightly raised and glassy-appearing (in sharp contrast with the unaffected skin), but after about a week of therapy the affected skin was so desiccated and flaking that therapy had to be discontinued (Figs. 4 and 5).

Additional treatments the patient has received for other conditions during the 15-year period include a 4-week course of amoxicillin–clavulanate and a 3-week course of cefuroxime, both of which were for perioperative management of chronic sinusitis. These antibiotic courses had no apparent effect on the erythematous scrotal skin. At the time of this manuscript the patient is 41 years old and continues to have well-demarcated erythema of the scrotal skin with the exact same borders as when the condition began.

## Discussion

This patient’s 15-year experience with RSS presents a compelling argument for a cutaneous microbial biofilm as the underlying etiology. The evidence in support of this conclusion can be summarized as follows:

- The symptoms began following a typical scenario that would facilitate biofilm formation. Vaginal secretions resting on the scrotum overnight combine a fluid environment containing microorganisms (both skin flora and vaginal flora) with sufficient time (e.g., 6–7 h) for biofilm formation to occur.
- The demarcation of affected skin closely following the hairline is readily explained by the hair follicles acting as scaffolding during biofilm formation. The hair follicles likely trapped fluid and held it in place on the skin surface overnight, with hairless skin being spared.
- The chronicity of the patient’s signs and symptoms as well as the recalcitrance of the condition to both topical and systemic antimicrobials is highly consistent with a biofilm infection.
- The persistence of gentian violet dye exactly matching the boundaries of the affected skin after quickly washing away from the unaffected skin strongly suggests, if not proves, the presence of abnormal material on the surface of the affected skin. The extracellular polysaccharide matrix of biofilm can avidly retain dye; therefore, the presence of a cutaneous microbial biofilm readily explains this observed phenomenon [20, 21]. The marked difference in response of the affected scrotal skin and unaffected scrotal skin to the application of 2.5% selenium sulfide, a known biofilm dispersal agent, further suggests the presence of biofilm [21].

The arguments against this conclusion can be mostly dismissed by a review of the biofilm literature and an acknowledgement of what is currently known and unknown about biofilm behavior:

- Any notion that biofilm formation cannot occur on intact skin is in conflict with current evidence. *Malasezzia furfur/ovale* has been shown to form biofilms both in vitro and in vivo, and cutaneous biofilm formation on intact skin is believed to be a major factor in the pathogenesis and chronicity of tinea versicolor [21, 22]. Biofilm formation on chronic wounds is widely recognized, and there is no proven attribute of intact epidermis that would prevent biofilm formation but is lacking on the surface of chronic wounds [18, 23, 24]. The intact scrotal skin may be particularly susceptible to biofilm formation due to its thin epidermis and irregular surface, as evidence shows that biofilms form more easily on rough surfaces [12].
- Visualization of biofilm is difficult using routine light microscopy and typically requires specialized staining or microscopy techniques (e.g., electron microscopy) [20, 25, 26]. Therefore, it is expected that the punch biopsy performed early in this patient’s disease course would appear essentially normal as evidence of a cutaneous microbial biofilm was not specifically sought.
- An argument could be made that the proposed mechanism of disease (i.e., vaginal secretions on the scrotum overnight) is likely such a common occurrence that the condition would be widespread and well-recognized by now. However, too little is known at this time to support such an argument. It is possible and even likely that specific parameters must be met (e.g., presence of particular microorganisms, host-specific deficiency in innate immunity) for cutaneous microbial biofilm formation to occur. Even further, the true incidence of RSS is almost certainly under-recognized as no large epidemiologic studies have been performed [5, 6]. Many affected patients may suffer with the condition for years without seeking treatment or discover their own symptomatic management independent of the medical community.
- An additional argument could be made citing doubt in how biofilm that is attached to the surface of the stratum corneum could a) cause discomfort and b) allow for the continued normal turnover of skin cells. Nerve endings are known to extend into and possibly slightly beyond the granulosum layer of the epidermis [27]. Given that biofilms are commonly 100 microns or greater in thickness and the scrotal stratum corneum is < 10 microns thick, it is reasonable that a biofilm could exert mechanical stimulus on epidermal nerve endings [28, 29]. It is also possible that biofilm releases chemical irritants into the skin or disrupts thermal regulation of the skin, causing the release of vasodilatory mediators that also interact with pain signaling. This would also explain the hypervascular appearance of the affected skin. Regarding normal skin cell turnover, biofilms are complex systems capable of utilizing nutrients and processing wastes [7, 15]. It is likely that biofilm is able to metabolize dead skin cells coming from the stratum corneum and allow for ongoing cell turnover.

In relating the findings in this case to other cases of RSS reported in the literature, a cutaneous microbial biofilm would seem to explain much of the reported disease burden. The majority of cases describe well-demarcated erythema with associated itching and burning that can last for years [1–3]. Details surrounding the onset of the condition are usually unclear, making it highly possible the condition unknowingly began after the patient failed to clean up following sex. The fact that the anterior scrotum is always affected also fits with this mechanism. Erythema closely following the distribution of hair is rarely specifically reported, though sparing of the hairless scrotal midline is commonly apparent in photographs. Nonetheless, it is possible that hair follicles may assist in biofilm formation but are not a necessary component. An association with prior topical corticosteroid use is often reported. It would seem the contribution of topical corticosteroids to the overall disease burden is likely overappreciated; however, it is possible that in some cases topical corticosteroids contribute to a favorable microbial or immunologic environment for biofilm formation to occur [30].

Psychiatric comorbidities are frequently reported with RSS (e.g., 75% prevalence in one small cohort of 12 patients) [2, 4]. Without a doubt, the emotional toll inflicted by chronic daily symptoms of scrotal itching and burning can be substantial. Unfortunately, in the absence of an evident organic disease process many clinicians likely consider the psychiatric comorbidities part of the cause of the symptoms rather than the more likely result of the symptoms, further worsening the situation for the patient. Successful symptomatic treatment of RSS has been reported with medications including gabapentin, amitriptyline, calcineurin inhibitors, and doxycycline [2, 5, 6, 31, 32]. The mechanism of successful treatment is largely unclear though it is likely to involve modification of neuropathic pain signaling (it is likely the 0.8% menthol powder is effective via a similar mechanism in this case) [33]. Whether or not the erythema resolves following these treatments is rarely specified, but it is often implied that the erythema remains despite improvement in symptoms (as occurred in this case).

Biofilms are often polymicrobial but given this patient’s history of tinea versicolor it would seem *Malassezia furfur/ovale* may be a crucial contributor to the pathology in this particular case, and the condition itself may represent a variant of tinea versicolor [21, 22, 34]. This patient’s recurrence of symptoms during daily ingestion of kefir may also suggest the involvement of *Lactobacillus* species, which are common components of normal vaginal flora. It is possible the abundant *Lactobacillus* organisms in kefir somehow triggered a temporary, heightened response to the biofilm [35].

Importantly, it stands to reason that a similar mechanism of cutaneous microbial biofilm formation may also underlie a significant portion of the disease burden of vulvodynia, a chronic condition characterized by vaginal itching and burning (and often erythema) that, like RSS, is in dire need of improved understanding and treatment options [36]. This suspicion is based on similarities with RSS in terms of both symptoms and chronicity, and a potential similar susceptibility of the vulvar skin to biofilm formation. Successful treatments for RSS have largely mirrored those for the more widely studied vulvodynia in that they are minimal and primarily focus on symptom relief via modification of neuropathic pain signaling. In addition, perhaps the only definitive treatment for vulvodynia involves surgical removal of the affected tissue, a characteristic therapy of last resort for biofilm infections [37, 38].

In conclusion, the probability that microorganisms, under proper conditions, can form biofilm on intact skin is poorly recognized. Hair covered portions of the genital skin may be particularly susceptible to this phenomenon. Further study is needed to confirm the proposed mechanism of cutaneous microbial biofilm formation as an underlying etiology of RSS, better characterize the prevalence of RSS, and further investigate both symptomatic and fully eradicative treatments. Informational campaigns to provide preventative hygiene recommendations to reduce future incidence, and to provide support for those currently affected are also needed. Finally, further investigation is needed into a similar mechanism of cutaneous microbial biofilm formation as a potential significant contributor to the disease burden of vulvodynia.

## Data Availability

Not applicable.
